# Macrophages in metaflammation – fueling chronic inflammation in metabolic disease

**DOI:** 10.1007/s00424-025-03141-0

**Published:** 2025-12-15

**Authors:** Ronja Kardinal, Dagmar Wachten

**Affiliations:** Institute of Innate Immunity, Biophysical Imaging, BMZ-II, Medical Faculty, University of Bonn, Venusberg-Campus 1, 53127 Bonn, Germany

**Keywords:** Metaflammation, Adipose tissue, Macrophages, Thermogenesis, Liver, Metabolic syndrome

## Abstract

Obesity is a leading global health issue, closely associated with a chronic low-grade inflammation termed metaflammation. Metaflammation is driven by immune cell reprogramming, particularly of macrophages. In white adipose tissue (WAT), obesity induces a shift from anti-inflammatory to pro-inflammatory macrophage phenotypes, contributing to insulin resistance and tissue fibrosis. Recent studies have also illuminated the role of macrophages in brown and beige adipose tissue (BAT and scWAT), where they influence thermogenic capacity. Beyond the adipose tissue, the liver is the other main metabolic organ impacted by obesity. Liver macrophages play a critical role in the pathogenesis of metabolic dysfunction-associated steatotic liver disease (MASLD) by promoting inflammation, lipid accumulation, and fibrosis. This review highlights the role of macrophages in the development and regulation of metaflammation in metabolic organs.

## Introduction

**Fig. 1:**
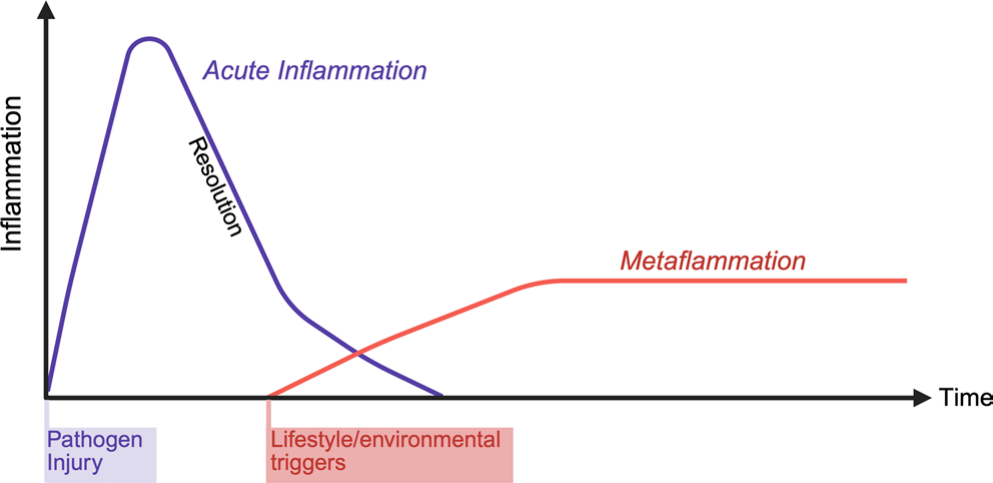
Inflammatory profile of an acute inflammation versus metaflammation. The y-axis represents the immune activation level, whereas the x-axis represents the time course how long the inflammation lasts. Created in BioRender. Wachten, D. (2025) https://BioRender.com/wbwxvvi

Over the last decades, obesity has become a global threat to public health. The WHO estimates that over half of all adults are overweight or obese, making obesity and its associated health consequences the most prevalent non-communicable disease (NCD) [1]. Excess caloric intake, resulting in weight gain and obesity leads to adipose tissue dysfunction, insulin resistance and diabetes, metabolic-associated fatty liver disease (MAFLD), atherosclerosis, and an increased risk of developing dementia [2, 3]. Combinatorial exposure to obesity-induced immunogenic compounds and lifestyle-related or environmental triggers can activate the immune system, leading to a low-grade, chronic inflammation termed metaflammation [2, 4]. Metaflammation differs markedly from acute inflammatory responses, as it lacks the typical clinical symptoms of acute inflammation (Fig. 1). Here, we will focus on the cellular changes in the immune system, specifically macrophages, in metabolic organs during obesity, promoting inflammation and the development of NCDs.

## Macrophages in the white adipose tissue – drivers of metaflammation in obesity

The white adipose tissue (WAT) is the central lipid storage of the body. WAT is also populated by immune cells, which undergo dramatic reprogramming during obesity. In the lean state, immune cells are polarized towards a type II immune environment, characterized by the secretion of the cytokines Interleukin (IL) −4, −10, −13, and transforming growth factor β (TGFβ) [2]. These resident immune cells include macrophages, dendritic cells (DC), innate lymphocytes type II (ILC2), eosinophils, CD4^+^ T helper type II cells (Th2), and regulatory CD4^+^ T-cells (Tregs) [5]. During obesity, infiltrating inflammatory macrophages increase the population of WAT-resident immune cells from 10% to 40%. This is mainly due to chemokine receptor 2 (CCR2)-dependent recruitment of classical monocytes, their local proliferation, and differentiation into inflammatory, monocyte-derived macrophages, leading to the histological appearing of crown-like structures (CLS), which form around hypertrophic adipocytes undergoing cell death [5]. Macrophage remodeling is accompanied by an overall shift towards a type I inflammatory environment, accompanied by a decrease of ILC2s, activation of DCs, and recruitment of Th1 and CD8^+^ T cells [5]. Thus, macrophages play a pivotal role in mediating inflammatory responses that disrupt metabolic homeostasis.

**Fig. 2:**
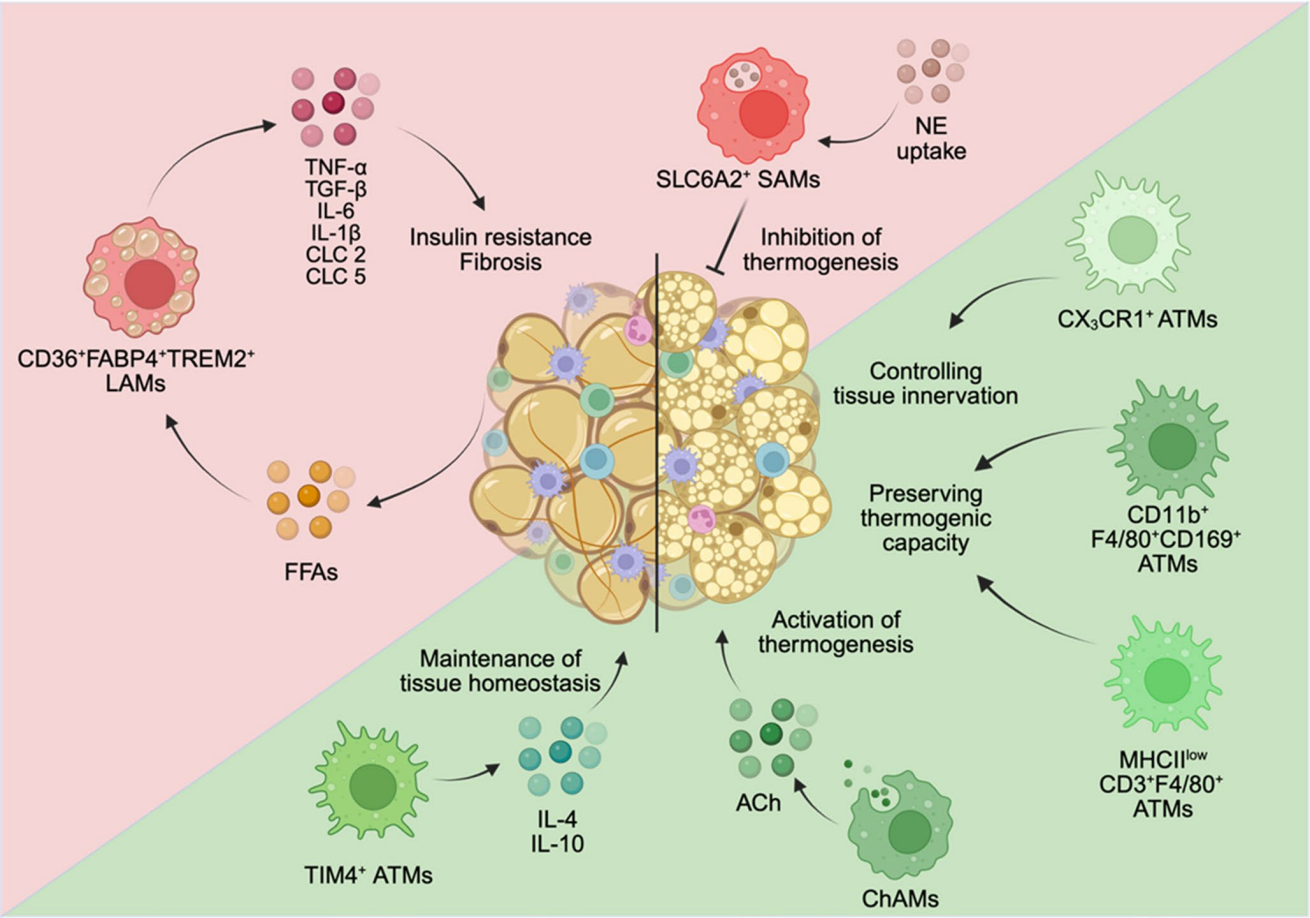
Overview macrophages in white, beige, and brown adipose tissue. Highlighted in red are the conditions promoting metaflammation, whereas the mechanisms that promote tissue homeostasis and function are highlighted in green. FABP4: Fatty acid binding-protein 4; TREM2: Triggering receptor expressed on myeloid cells 2; LAMs: Lipid-associated macrophages; FFA: Free fatty acids; ATMs: Adipose tissue macrophages; ACh: Acetylcholine; ChAMs: cholinergic adipose macrophages; NE: Norepinephrine; SAMs: Sympathetic neuron-associated macrophages. Created in BioRender. Wachten, D. (2025) https://BioRender.com/to3m1yt

Adipose tissue macrophages (ATMs) in WAT are highly specialized, distinguished by surface phenotype, origin, and function. Generally, yolk sac-derived Tim4^+^ macrophages maintain WAT homeostasis, whereas monocyte-derived macrophages infiltrating the WAT during obesity are mainly inflammatory (Fig. 2) [6–8]. The recruited monocyte-derived macrophages adopt a lipid-associated phenotype, leading to the term lipid-associated macrophages (LAMs), characterized by high expression of lipid-handling genes such as CD36, FABP4, and TREM2 (Triggering receptor expressed on myeloid cells 2) [8, 9]. Despite their role in lipid clearance, LAMs also exhibit pro-inflammatory properties, contributing to the chronic inflammatory state of obese adipose tissue. Within the CLS surrounding necrotic adipocytes, macrophages secrete pro-inflammatory cytokines such as TNF-α, IL-6, and IL-1β, which impair insulin signaling in adipocytes and exacerbate systemic insulin resistance (Fig. 2) [5, 10–12]. The activation of pattern recognition receptors, such as TLR4, by free fatty acids (FFAs) further amplifies macrophage-driven inflammation, leading to a self-perpetuating cycle of metabolic dysfunction (Fig. 2) [13, 14]. ATMs are also central to fibrosis development in obese adipose tissue by secreting profibrotic cytokines such as TGF-β, which promote extracellular matrix deposition and impair adipose tissue flexibility. Fibrotic adipose tissue exhibits reduced insulin sensitivity and increased inflammation, reinforcing the progression of metabolic dysfunction-associated diseases (Fig. 2) [15].

## Changes in cellular metabolism in macrophages and its role in metaflammation

In homeostatic WAT, macrophage metabolism relies on fatty acid oxidation and mitochondrial respiration, sustaining the production and secretion of IL-4 and IL-10 [16, 17]. During obesity, ATMs are characterized by increased glycolysis and oxidative phosphorylation (OX PHOS), altering the levels of oxidized phospholipid (OxPL) species that regulate gene expression and cellular metabolism [18]. However, whether these ATMs originate from the yolk-sac or the bone marrow has not been fully addressed yet. A decreased oxygen consumption rate and mitochondrial respiration, as well as activation of the pentose phosphate pathway results in an increased production of reactive oxygen species (ROS) and nitric oxide (NO). In conjunction with these processes, oxidized LDL production or cholesterol crystals activate the NLRP3 inflammasome, leading to the expression of IL-1β and inducing a type I inflammatory macrophage phenotype during metaflammation [4]. Thus, reprogramming of macrophage metabolism is key to induce metaflammation [19].

## Brown and beige adipose tissue – emerging targets in obesity treatment

Whereas WAT primarily functions as a lipid storage with endocrine properties and is the main driver of metaflammation, brown AT (BAT) and beige AT fulfill different roles. BAT develops and differentiates prenatally to protect most newborn mammals from a cold environment after birth through heat production via non-shivering thermogenesis (NST) [20]. The prevalence of active BAT in human adults greatly varies and depends on a plethora of factors including age and BMI [21–26]. BAT is a highly innervated tissue composed of mainly multi-locular brown adipocytes with a high mitochondrial content. Brown adipocytes dissipate heat via NST after sympathetic neurons release norepinephrine (NE) in response to cold exposition. NE activates β-adrenergic receptors (ARs), triggering a signaling cascade that activates lipolysis and the release of FFAs from triglyceride (TG) stores. In turn, the thermogenic uncoupler, uncoupling protein 1 (UCP1), is activated, severing cellular respiration from ATP synthesis by transporting H^+^ ions across the inner mitochondrial membrane (IMM) [20, 27]. Thereby, the excess energy dissipates as heat. Next to classic brown adipocytes, beige adipocytes (brite, brown-in-white, brown-like) derive from subcutaneous WAT (scWAT) in response to different environmental, pharmacological, or genetic stimuli. Beige adipocytes are characterized by the expression of thermogenic genes including *UCP1*, as well as a higher mitochondrial content, and smaller lipid droplets compared to classic white adipocytes [28–30]. A higher prevalence of BAT is linked to a decrease of central obesity, which in turn contributes to the metabolic benefits of BAT activity [26]. Thus, activating BAT and scWAT browning or beiging to target the obesity pandemic and improve obesity and metabolic diseases has become a key objective in the field.

Apart from its well-known function as a thermogenic organ, BAT promotes metabolic health by acting as a metabolic-sink for glucose, branched-chain amino acids (BCAAs), and lipids [31–33]. Thus, increasing whole-body energy expenditure through NST is not the only benefit of BAT activation on metabolic health. During NST, brown adipocytes oxidize glucose taken up through glucose transporter type 1 and 4 (GLUT-1, 4) [20, 34], improving glucose tolerance and insulin sensitivity [35]. Furthermore, BAT transplantation attenuates inflammatory gene expression and the development of insulin resistance in WAT [36]. While, glucose uptake into BAT after cold exposure is increased in healthy adults, it is impaired in individuals with obesity, supporting the notion that BAT activity and volume should be targeted under pathological conditions like obesity [32].

To maintain its thermogenic function and restore intracellular FFA levels, BAT is taking up FFAs and monoacylglycerol hydrolyzed from circulating TGs by lipoprotein lipase (LPL). This makes BAT an important lipid-plasma clearing organ in rodents, responsible for up to 50% of total TG disposal after prolonged cold exposure [31, 37, 38]. Increased plasma TG and FFA concentrations are linked to the development of insulin resistance, coronary heart disease, and T2D [39, 40]. Notably, very-low-density lipoprotein TG (VLDL-TG) and FFA clearance rates are higher in overweight and obese women with high BAT volume. However, the small amount of active BAT in the study participants per se, responsible for < 1% of total TG disposal, suggests a role of other organs enhancing lipid-plasma clearance [21].

BCAA catabolism is increasingly recognized as a key player in the development of obesity and metabolic disorders. Elevated plasma BCAA levels are associated with insulin resistances in individuals with T2D [41]. Upon cold exposure, BAT promotes systemic BCAA clearance to utilize BCAAs in the mitochondria during NST. BCAA catabolism in BAT mitochondria is mediated by SLC25A44, a BCAA carrier required for mitochondrial BCAA import and subsequent oxidation in the mitochondrial matrix [33]. BCAA catabolism is crucial to uphold BAT NST, as it is producing mitochondrial amino acids and tricarboxylic acid (TCA) cycle intermediates [33, 42, 43]. Thus, individuals presented with obesity and the metabolic syndrome might profit from BAT activation followed by systemic BCAA clearance.

Taken together, BAT activation presents a potent target to tackle obesity and metabolic disturbance beyond the borders of energy dissipation. Identifying BAT activity modulators becomes crucial for future drug and treatment development.

## The role of macrophages in brown and beige adipose tissue

BAT harbors a plethora of immune cell populations, including monocytes and macrophages, T cells, B cells, ILCs, NK cells, and DCs [44, 45]. In recent years, the role of immune cells in controlling the thermogenic function of brown and beige adipocytes has been extensively studied. ATMs regulate thermogenesis by modulating sympathetic nervous system activity [46]. The BAT macrophage pool contains two distinct macrophage subsets, which are either CD206^+^ or CD226^+^ [44]. CD206^+^ macrophages originate from the bone marrow, whereas CD226^+^ macrophages are yolk sac-derived [47]. During obesity, macrophages shift toward a phenotype that inhibits beige adipogenesis and promotes ‘whitening’ of thermogenic adipose tissue, causing the formation of unilocular lipid droplets, accompanied by the loss of thermogenic gene signature [48]. The decrease of UCP1 expression is linked to an increase in pro-inflammatory, macrophage-derived cytokines like TNFα, IL-1β, C-C motif chemokine ligand CCL2 and CCL5 [49–51]. Recently, a distinct CD36^+^ subset of LAMs derived from pro-inflammatory monocytes have been identified that promote the loss of brown adipocyte identity in obese BAT [52]. Under conditions of metabolic overload, brown adipocytes dispose mitochondrial components in extracellular vesicles (EVs) that are cleared by CD36^+^ LAMs through action of the CD36 scavenger receptor. Interestingly, LAMs treated with these EVs release TGFβ1, which in turn increases aldehyde dehydrogenase 1 family member A1 (ALDH1A1) in brown adipocytes. ALDH1A1 is associated with the loss of brown fat identity, while *Aldh1a1* knock-out leads to increased mitochondrial gene expression [52]. These novel findings delineating BAT responses during obesity on a single-cell level highlight the potential to target ATMs to prevent BAT whitening.

Another subset of macrophages prevents NE-mediated browning of WAT. The so-called sympathetic neuron-associated macrophages (SAMs) express the NE transporter Slc6a2, which conveys NE import, followed by its degradation via the monoamine oxidase (MAOa) (Fig. 2). SAMs display a pro-inflammatory profile already at a steady state, caused by either adrenergic signaling or NE import. In the obese state, SAMs are recruited to WAT and activated, expressing pro-inflammatory cytokines like TNFα. Interestingly, loss of *SLC6a2* in SAMs rescues the obesity-induced hypertrophy of white and brown adipocytes already at ambient temperatures and increases serum NE levels as well as WAT browning after acute cold exposure in obese mice [53]. Targeting the function of a single subset of macrophages to elevate NE levels in AT could improve the metabolic state during obesity by promoting adaptive thermogenesis and lipolysis.

While SAMs and LAMs have a detrimental effect on thermogenic adipose function, alternatively activated macrophages in BAT and scWAT promote a thermogenic response through catecholamine secretion in an IL-4-dependent manner. An acute cold challenge increases the expression of CD206, CD301, and arginase (ARG1) in BAT and scWAT macrophages, and leads to the secretion of NE accompanied by an increase in energy expenditure [54]. Furthermore, cholinergic adipose macrophages (ChAMs) contribute to the thermogenic response in scWAT in a non-neuronal cholinergic circuitry by secretion of acetylcholine. ChAMs express choline acetyltransferase (ChAT) and accumulate after cold exposure in scWAT, promoting thermogenesis by activating the cholinergic receptor nicotinic alpha 2 subunit (CHRNA2) uniquely expressed in beige adipocytes (Fig. 2) [55].

Apart from directly improving the thermogenic response, macrophages also control the innervation of BAT [46]. CX_3_CR1^+^ macrophages expressing the nuclear transcription regulator methyl-CpG binding-protein 2 (MECP2) maintain sympathetic innervation via plexin-semaphorin macrophage-neuron crosstalk to ensure sufficient NE release into BAT during cold exposure (Fig. 2) [46]. Furthermore, CD11b^+^F4/80^+^CD169^+^ BAT macrophages increase after cold exposure and phagocytose EVs containing damaged mitochondrial particles, which ensures optimal NST [56], whereas MHCII-low, CD3^+^F4/80^+^ macrophages increase during chronic β-adrenergic stimulation in BAT, preserving thermogenic capacity (Fig. 2) [57]. How brown adipocytes contribute to macrophage accumulation and, thereby, ensure an optimal thermogenic response, is not well understood. In response to cold, brown adipocytes increasingly express and secrete CXCL12. Apart from functioning in an autocrine manner, controlling brown adipocyte activation upon NE stimulation, CXCL12 exerts paracrine functions in controlling the recruitment and retention of monocyte-derived macrophages into BAT [58].

## Therapeutic targeting of macrophages in thermogenic fat

The great heterogeneity of AT macrophages, as well as their capacity to control thermogenic processes positively or negatively, makes them a vigorous target for future obesity treatments. A recent study suggests the use of nanoparticle (NP)-mediated immunomodulation of macrophages in AT to tackle obesity-related inflammation and metabolic dysfunction [59]. The administration of apigenin (Api), a plant flavonoid, through Api-coated NPs to AT reduced inflammatory gene expression and reprogrammed AT macrophages into an anti-inflammatory profile. Furthermore, Api-NP administration reduced body weight and AT mass. These metabolically favorable effects are caused by the increased expression of thermogenic markers (*UCP1*,* CIDEA*,* PRDM16*) in white adipocytes and in turn the browning of WAT in obese mice [59]. Another notable study presents how UCP1 expression can be induced in AT macrophages. The lack of hypoxia-inducible factor-1 (HIF1) results in UCP1 expression in macrophages, independent from cold- or β-adrenergic stimulation [60]. While UCP1-expressing macrophages in AT were reduced with increasing BMI, an accumulation of UCP1-producing CD163^+^ macrophages in scWAT of cold exposed individuals was involved in browning of the tissue [60, 61]. Taken together, these findings highlight the importance of understanding the thermo-regulatory mechanisms of AT macrophages, to progress in preventing and treating obesity, metaflammation, and metabolic syndrome.

## Metaflammation beyond the adipose tissue – liver macrophages as mediators of MASLD progression

**Fig. 3:**
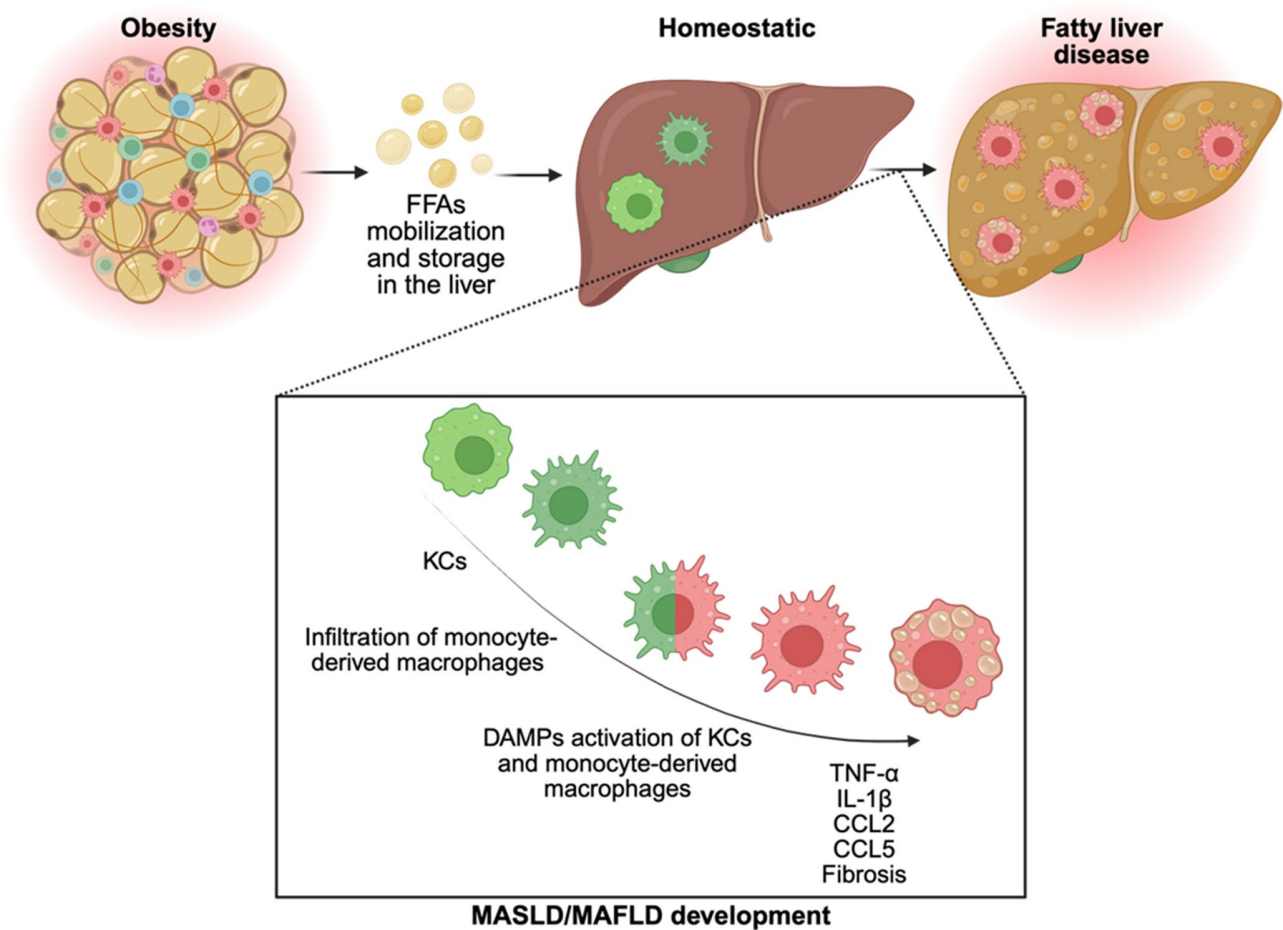
Beyond the adipose tissue – metaflammation in the liver. When the capacity of storing lipids in the adipose tissue is reached and insulin resistance has developed, free fatty acids (FFAs) are mobilized, increasing the flux to and storage in the liver. This is accompanied by macrophage reprogramming, particular of Kupffer cells (KCs) and the infiltration of monocyte-derived macrophages, promoting the development of fatty liver diseases. MASLD: metabolic dysfunction-associated steatotic liver disease; MAFLD: metabolic dysfunction-associated fatty liver disease; DAMPs: Damage-associated molecular pattern. Created in BioRender. Wachten, D. (2025) https://BioRender.com/to3m1yt

Beyond adipose tissue, the liver is the other main metabolic organ impacted by obesity. When the capacity of storing lipids in the adipose tissue is reached and insulin resistance has developed, FFAs are mobilized, increasing the flux to and storage in the liver [62]. This contributes to the development of metabolic dysfunction-associated fatty liver disease (MAFLD), which is widely regarded as the hepatic manifestation of the metabolic syndrome (Fig. 3) [63]. MAFLD has been redefined to metabolic dysfunction-associated steatotic liver disease (MASLD), offering a more inclusive framework for diagnosis and research that acknowledges the overlap with alcoholic liver disease, requires only one cardiometabolic risk factor for diagnosis, and allows better identifying lean individual with fatty liver disease [64].

Metaflammation also occurs in the liver and has been shown to promote progression of the metabolic syndrome. The liver harbors a heterogeneous population of macrophages. Here, we focus on the Kupffer cells (KCs). The liver is the first organ during development that contains tissue-specific macrophages, the KCs [65], and only later, monocyte-derived macrophages infiltrate, e.g., in response to metabolic stress [66–68]. After extensive periods of diet-induced obesity and MAFLD, KCs lose self-maintenance and are replenished by monocyte-derived KC-like cells, further increasing the heterogeneity in the tissue [66, 68, 69], and, thereby, progression of MAFLD. Liver macrophages are highly responsive to lipid accumulation and hepatocyte stress. Hepatocytes undergoing lipotoxic damage release damage-associated molecular patterns (DAMPs) that activate KCs and infiltrating macrophages via pattern recognition receptors such as TLR4 and NLRP3 inflammasomes [70]. This leads to the production of inflammatory cytokines (TNF-α, IL-1β) and chemokines (CCL2, CCL5), further recruiting inflammatory monocytes to the liver (Fig. 3).

LAMs in the liver share similarities with their adipose tissue counterparts, expressing high levels of TREM2, CD9, and GPNMB (glycoprotein NMB) [8, 66]. These cells localize to steatotic and fibrotic regions of the liver, where they regulate lipid clearance and tissue remodeling [71]. TREM2 deficiency exacerbates MASLD, leading to mitochondrial dysfunction and impaired fatty acid oxidation in hepatocytes [72]. Despite their role in lipid handling, LAMs also contribute to fibrosis by interacting with hepatic stellate cells (HSCs) and secreting profibrotic factors [73].

## Conclusion

In conclusion, obesity-induced metaflammation represents a complex interplay between metabolic dysfunction and immune dysregulation, driven by the dynamic behavior of immune cells, in particular of macrophages across various tissues. In white adipose tissue, the shift from homeostatic to inflammatory macrophage populations underpins the chronic inflammatory environment that disrupts metabolic homeostasis and promotes insulin resistance, fibrosis, and systemic disease. Brown and beige adipose tissues, once considered passive players, have emerged as crucial regulators of energy expenditure and systemic metabolism, with their function tightly regulated—positively and negatively—by distinct macrophage subsets. Furthermore, the liver, as a central metabolic hub, exhibits its own macrophage-mediated inflammatory response, contributing to the progression of metabolic-associated steatotic liver disease. The remarkable heterogeneity and plasticity of tissue macrophages present both a challenge and an opportunity: targeted immunomodulation, whether through nanoparticle delivery systems or genetic manipulation, offers a promising avenue to not only mitigate inflammation but also restore thermogenic and metabolic functions. As our understanding of these processes deepens, macrophages may become pivotal therapeutic targets in the battle against obesity, metaflammation, and their associated non-communicable diseases.

## Data Availability

Not applicable.

## Abbreviations

ALDH1A1: Aldehyde dehydrogenase 1 family member A1
API: Apigenin
ARG1: Arginase
Ars: Adrenergic receptors
AT: Adipose tissue
ATMs: Adipose tissue macrophages
ATP: Adenosine triphosphate
BAT: Brown adipose tissue
BCAAs: Branched-chain amino acids
BMI: Body mass index
CCL: C-C motif chemokine ligand
CCR2: Chemokine receptor 2
ChAMs: Cholinergic adipose macrophages
ChAT: Choline acetyltransferase
CHRNA2: Cholinergic receptor nicotinic alpha 2 subunit
CIDEA: Cell death-inducing DFFA like effector a
CLS: Crown-like structures
DAMPs: Damage-associated molecular patterns
DC: Dendritic cell
EVs: Extracellular vesicles
FABP4: Fatty acid binding-protein 4
FFAs: Free fatty acids
GLUT: Glucose transporter
GPNMB: Glycoprotein NMB
HIF1: Hypoxia-inducible factor-1
HSCs: Hepatic stellate cells
IL: Interleukin
ILC2: Innate lymphocytes type II
IMM: Inner mitochondrial membrane
KCs: Kupffer cells
LAMs: Lipid-associated macrophages
LDL: Low-density lipoprotein
LPL: Lipoprotein lipase
MAFLD: Metabolic dysfunction-associated fatty liver disease
MAOa: Monoamine oxidase
MASLD: Metabolic dysfunction-associated steatotic liver disease
MECP2: Methyl-CpG binding-protein 2
NCD: Non-communicable disease
NE: Norepinephrine
NK: Natural killer cells
NLRP3: NOD-,LRR-and pyrin domain-containing protein 3
NO: Nitric oxide
NP: Nanoparticle
NST: Non-shivering thermogenesis
OX PHOS: Oxidative phosphorylation
OxPL: Oxidized phospholipid
PRDM16: PRD1-BF1-RIZ1 homologous domain containing 16
ROS: Reactive oxygen species
SAMs: Sympathetic neuron-associated macrophages
scWAT: Subcutaneous white adipose tissue
TCA: Tricarboxylic acid
TG: Triglycerides
TGFβ: Transforming growth factor β
Th: T helper cells
TLR4: Toll-like receptor 4
TNF-α: Tumor necrosis factor α
Tregs: Regulatory T cells
TREM2: Triggering receptor expressed on myeloid cells 2
T2D: Type 2 diabetes
UCP1: Uncoupling protein 1
VLDL-TG: Very-low-density lipoprotein triglyceride
WAT: White adipose tissue
